# Injury and death to armored passenger-vehicle occupants and ground personnel from explosive shock waves

**DOI:** 10.1038/s41598-023-29686-7

**Published:** 2023-02-13

**Authors:** David C. Viano

**Affiliations:** ProBiomechanics LLC, 265 Warrington Road, Bloomfield Hills, MI 48304 USA

**Keywords:** Risk factors, Engineering

## Abstract

This study evaluated the risks for injury and death to occupants from blast waves to the side and underbody of an armored passenger-vehicle and to ground personnel from free-field blast waves. The Kingery-Bulmash empirical relationships for explosive shock waves were augmented by the Swisdak empirical relations for stand-off distances up to Z = 39.8 m/kg^1/3^ to tabulate shock-wave characteristics using the Friedlander wave-shape. A 15 kg, hemispherical explosion was analyzed in detail for the shock wave velocity and compression of air behind the wave front. An armored SUV was analyzed with Z = 1.6 m/kg^1/3^ (4 m) standoff distance from pressure loading on the near-side, far-side and underbody. The rigid body displacement was 0.36 m and 7.8° yaw for a side loading. When a segment of the occupant compartment accelerates inward, there are risks for injury from the intrusion. Energy is transferred to the occupant by deformation of their body (E_d_) and by velocity increasing the kinetic energy of the body region (E_k_). Body deformation injures an occupant by exceeding the tolerable compression (crush mechanism) or exceeding the rate-dependent tolerance, which is defined by the rate times the extent of compression (viscous mechanism). The risk for injury and death to ground personnel was analyzed for free-field blast waves by stand-off distance and TNT weight. A 15 kg charge posed a 99% risk of death at 3.9 m, 50% risk at 5.2 m, 1% risk at 7.8 m and injury threshold at 8.2 m. A 100 kg charge posed a 99% risk of death at 8.5 m, 50% risk at 11.6 m, 1% risk at 17.3 m and injury threshold at 18.0 m. The study describes the steps to analyze blast loading of an armored passenger-vehicle for risks of occupant injury. It describes the steps to analyze injury risks to ground personnel from blast wave pressure.

## Introduction

Over the past 20 years, there has been an increase in the use of road-side bombs and buried explosives to injure vehicle occupants and ground personnel^1–4^. This spawned the development of armored passenger-vehicles to protect occupants from the effects of blast overpressure, fragments and bullets.

Blast testing of armored passenger-vehicles is common today. It often involves an explosive charge positioned to the side of the vehicle with stand-off distances of 2–4 m using 15 kg TNT equivalent explosive placed above ground^5^. Other tests involve 6–10 kg buried charges under the occupant compartment or wheel of the vehicle. In many cases, an ATD (anthropometric test devices) is seated in the occupant compartment to assess risks for injury and death inside the armored passenger-vehicle. The most commonly used ATD is the Hybrid III dummy, which is fit more than 100 channels to assess risks in different body regions^6,7^.

An understanding of blast over-pressure characteristics with stand-off distance is needed to develop adequate armor and glass recognizing there are trade-offs with the amount of armor that can be fit on the vehicle because of the GVW (gross vehicle weight) weight limit of production vehicles. Part of the basis for today’s understanding comes from instrumented blast tests.

### Blast test data

Kingery^8^ reported on a series of multi-ton TNT explosion experiments starting in 1959 at the Suffield Experimental Station (SES), Canadian. A blast line was instrumented to measure the pressure–time history of the blast wave at radial distances from the explosion. A 5-ton TNT test was conducted in 1959, 20-ton test in 1960, 100-ton test in 1961 and 500-ton test in 1964. The TNT was configured in a hemisphere with the flat side resting on the ground. The TNT had a density of 1500 kg/m^3^ and was cast in 30×30×10 cm blocks weighing 14.8 kg.

The blast line data for four tests was processed for peak incident and reflected pressure (P_i_ and P_r_), incident and reflected impulse (I_i_ and I_r_), arrival time (t_a_), duration of the positive pressure pulse (t_o_) and shock wave velocity (U). The velocity (U) of the shock front was calculated from the arrival time (t_a_) and distance (R) from the center of the explosion (R_0_ = 0) with U = R/t_a_. The velocity of the shock front was used to calculate the peak overpressure (P_i_) from the Rankine-Hugoniot relationship with

1 $$ {\text{P}}_{{\text{i}}} = \gamma {\text{P}}_{0} \left( {{\text{U}}/{\text{C}}_{0} } \right)^{{2}} $$

where γ is the ratio of specific heats, P_0_ is the pressure and C_0_ is the speed of sound in ambient air^9–11^. For ambient conditions, γ = 1.401, P_0_ = 101.325 kPa and C_0_ = 340 m/s. The positive pressure duration (t_0_) is the time between the shock arrival (t_a_) and the end of the positive pressure. The positive duration was difficult to consistently and repeatably measure, so various methods were used to provide an accurate duration. Cube root scaling and altitude corrections were used to adjust the data to sea level conditions and 0.45 kg (1 lb) charge. Kingery and Bulmash^12^ further analyzed the data and provided relationships for the shock-wave characteristics.

### Cube root scaling

The Hopkinson–Cranz law is based on empirical observations and describes a scaled distance (Z):

2 $$ {\text{Z}} = {\text{R}}/{\text{W}}^{{{1}/{3}}} $$

where R is the distance from the detonation center (R_0_ = 0) and W is the mass of the charge in kg equivalent TNT^13^. Z is a dimensional variable in m/kg^1/3^. The law indicates that similar shock waves are generated by two different explosions at the same scaled distance Z, assuming similar ambient pressure and temperature. Sachs^14^ improved the Hopkinson-Cranz law by including the effect of atmospheric conditions in the scaled distance, so that Z = R(P_0_/W)^1/3^, where P_0_ is the ambient pressure. Baker et al.^15^ performed a more complete dimensional analysis of explosive shock waves. Wei and Hargather^16^ extended the scaling to include under-sea and air explosions in a comprehensive study.

The scaled distance (Z) is used to characterize shock waves as they propagate. The Kingery and Bulmash^12^ curves are often used to describe the shock wave. The relationships are based on the Kingery ^8^ tests and include the blast wave incident and reflected pressure (P_i_ and P_r_), incident and reflected impulse (I_i_ and I_r_), shock wave velocity (U), arrival time (t_a_) and duration of the positive pressure pulse (t_o_) from analysis of the Suffield blast testing using the dimensional scaled distance Z. The empirical relationships are the basis for most analyses of blast responses. Swisdak^17^ provided simplified empirical relationships, which closely approximated the original Kingery-Bulmash data.

Equation (2) is widely used in the study of blast waves. A modification is needed for different types and placement of explosive charges with Z = R/(βW^1/3^), where β is a constant that depends on a spherical, hemispherical or shaped charge and different placements. For example, if β = 1.0 for a spherical charge, β = 1.8–2.0 for a hemispherical charge and β > 2.0 for shaped charges. If symmetry is assumed between a hemispherical and spherical charge, a doubling of the charge weight is predicted between spherical and hemispherical charges (β = 2.0). Gan et al.^18^ compared pressure at relatively large stand-off distances and found that β = 2 was accurate. Other blast testing found ground effects influenced the hemispherical shock wave and β = 1.8 was more reasonable closer to the detonation^19,20^. The height of the charge above ground is another factor. Omang et al.^21^ analyzed the influence a charge height above ground.

There are many studies simplifying the Kingery-Bulmash relationships that rely on different scaling techniques and methods^22–31^. Dewey, McMillan^23^ provided a compendium of blast wave properties. Jankura et al.^24^ reviewed various simplifications and compared eight different models for 0.1, 0.5 and 1.0 kg TNT explosions. There were large differences among the models. The average difference and standard deviation were 52% ± 52% using the Kingery-Bulmash estimates as the gold standard. A 0.1 kg TNT explosion was also evaluated at distances of 1, 2 and 5 m, again with large differences between different models. Goel et al.^28^ compared five empirical relationships including Kingery-Bulmash. Again, differences were observed. Cormie et al.^32^ analyzed scaled distance relationships near the detonation and provided revised relationships. Bogosian et al.^33^ considered uncertainty in various simplified models. Anas et al.^34^ reviewed other scaling studies and also reported differences. This is consistent with other evaluations^35^.

Modern explosive testing generally confirms that the Kingery-Bulmash relationships provide a useful description of shock waves as they propagate. There are some exceptions^36^. Figuli et al.^25^ studied experimental and numerical responses with different explosives and evaluated TNT equivalency. There are a number of numerical simulations for shock wave propagation and reflection off objects^37–40^. These provide additional insights for objects in the path of propagation. There are many computational tools with varying fidelity related to responses of vehicles and occupants subjected to shock waves. Shin et al.^29^ compared incident and reflected pressure with spherical explosions in an effort to improve CFD numerical simulations.

Dewey^20^ provided a blast calculator in Excel for the properties of blast waves from surface-burst TNT explosions. The calculations are based on the AirBlast program. The pressure results are close to those given by the online UN SaferGuard calculator, which is based on the Kingery-Bulmash parameter calculator^41^. For this study, the Kingery-Bulmash and Swisdak relationships were considered sufficient to define the pressure loading of an armored passenger-vehicle and ground personnel at stand-off distances Z > 0.4 m/kg^1/3^.

### Injury assessment for armored passenger-vehicle occupants and ground personnel

It is useful to have simple methods to give first-order effects of shock waves on the vehicle and occupants and ground personnel. This type of analysis can direct further considerations for risks to occupants from rigid body displacement of the vehicle, local deformation of sheet metal and structures into the occupant compartment and ground personnel in the vicinity of the vehicle. Local displacements of the interior can involve high velocity toward the occupant with the segment springing back into position after the pressure passes. Local deformations load the occupant’s feet in contact with the floor. Other areas of the interior can be deformed into the occupant.

The data linking the Kingery-Bulmash and Swisdak empirical relationships for the shock wave and injury risks to vehicle occupants and ground personnel is the pressure and duration of the shock wave. The pressure depends on the stand-off distance, size of charge and other factors. The pressure can directly injure ground personnel and indirectly occupants inside an armored passenger-vehicle. This study lays out the connections between the data and the evaluation of injury risks. The aims of the study are to describe the steps to analyzed blast loading of an armored passenger-vehicle and determine risks for occupant injury and to describe the risks for ground-personnel injury from the propagation of blast waves.

## Methods

### Empirical data on hemispherical blasts

Kingery et al.^42^, Kingery and Pannill^43^, Kingery^8^ and Kingery and Bulmash^12^ described blast testing that is the basis for most empirical relationships for incident and reflected pressure (P_i_ and P_r_), incident and reflected impulse (I_i_ and I_r_), shock wave velocity (U), arrival time (t_a_) and duration of the positive pressure pulse (t_o_). Swisdak^17^ provided simplified empirical relationships, which closely approximated the original data. The blast tests involved spherical and hemispherical explosions with pressure measurements at standoff distance (R) from the center of the blast (R_0_). The testing confirmed a dimensional parameter (Z) that predicted shock wave pressure with different weights (W) of the explosive charge and stand-off distance (R) with Z defined as Z = R/(W^1/3^) from Eq. (2).

The empirical relationships for blast waves were tabulated for Z = 0.05–40 m/kg^1/3^, which represents R = 0.123–98 m for a 15 kg TNT hemispherical charge. Not all parameters of the blast wave can be calculated by a particular empirical formula. Several sources were used to tabulate the shock wave characteristics. Most of the results have been confirmed against testing from low-weight to nuclear explosions, although caution has been raised that not all parameters are accurately determined by the empirical relationships for small Z^16,18^.

### Explosions and shock waves

The explosion involves a chemical reaction that converts the charge weight into heat, fragments and shock wave. TNT (TriNitroToluene) has an energy (E) equivalence of 62,760 kJ/15 kg charge weight. When ignited, TNT (C_7_H_5_N_3_O_6_) decomposes into nitrogen, carbon dioxide, water and carbon with 4C_7_H_5_N_3_O_6_ → 6N_2_(g) + 7CO_2_(g) + 10H_2_O(g) + 21C(s).

Various estimates have been made on the amount of energy converted into the shock wave. The explosion is a chemical reaction that occurs at high temperature with combustion by products, fragments and a shock wave. Shin et al.^44^ found with sufficiently high temperature > 1800 K, most of the combustion reaction occurs. Taylor^9,10^ estimated that as much as 45% of the energy goes to heating the air and is not available to do work. The detonation velocity of the explosive is the speed of the chemical reaction that forms the shock wave. For TNT, the detonation velocity is U = 6,950 m/s with variations depending on the density, shape and other factors of the charge^45,46^. The detonation speed of C4 is U = 8,092 m/s. The speeds of other explosives have been reported^47,48^. TNT equivalence factors are available^49,50^.

The shock wave from an explosion propagates radially in a shell of high pressure with increasing radius from the center of the explosion (R_0_ = 0). The stand-off distance (R) is the radius of the shell at the wave front. Since the radius increases with time, the surface area of the shell and volume inside the wave front increase with time and stand-off distance. The volume (V) behind the expanding front of the shock wave is V = 4πR^3^/3. The surface area (A) of the shock wave front is A = 4πR^2^.

The air in front of the shock wave is at ambient pressure and is displaced outward by the shock wave. This compresses the air, because the velocity of the shock wave is greater the speed of sound in air. The weight (W_t_) of the air compressed behind the wave front is the weight of the volume of air at that radius (R) under ambient conditions with W_t_ = ρ_0_V, where ρ_0_ is the density of air (1.222 kg/m^3^) at ambient pressure (P_0_ = 101.325 kPa) and temperature at sea level. The weight of air compressed behind the shock is progressively packed up to the wave front of the expanding shell.

### Friedlander shock wave

The shape of the pressure behind the shock front is defined by an exponential decay in pressure based on Friedlander^51^. The shock-wave pressure (P) is:

3 $$ {\text{P}} = {\text{P}}_{{{\text{max}}}} \left( {{1} - {\text{t}}/{\text{t}}_{0} } \right){\text{e}}^{{ - {\text{bt}}/{\text{t0}}}} $$

where P_max_ is the pressure at the wave front, t is time after the shock arrival (t_a_) with t = T—t_a_, where T is the time from detonation. The duration of the positive pressure pulse is t_0_ and b is a parameter describing the decay of pressure behind the wave front. The pressure is reported as the incident pressure (P_i_) in the free-field or the reflected pressure (P_r_). The reflected pressure assumes the wave hits a flat, rigid surface as it propagates. The amount of energy in the shock wave is related to the impulse (I) of the wave shape, which is the integral of the pressure from the shock wave arrival (t_a_) to the cross-over in pressure (t_a_ + t_o_) with t_0_ the positive pulse duration. The incident impulse is:

4 $$ {\text{I}}_{{\text{i}}} = {\text{P}}_{{\text{i}}} {\text{t}}_{0} \left[ {{1}/{\text{b}}{-}\left( {{1} - {\text{e}}^{{ - {\text{b}}}} } \right)/{\text{b}}^{{2}} } \right] $$

The impulse is reported as the free-field or incident impulse (I_i_) or the reflected impulse (I_r_). The ratio of the impulse and pressure (I_i_/P_i_) is related to t_0_ and b:

5 $$ {\text{I}}_{{\text{i}}} /{\text{P}}_{{\text{i}}} = {\text{t}}_{0} \left( {{\text{b}} - {1} + {\text{e}}^{{ - {\text{b}}}} } \right)/{\text{b}}^{{2}} $$

The Kingery-Bulmash and Swisdak empirical relationships give P_i_, I_i_ and t_0_, so the b-value can be calculated for different stand-off distances and charge weights. The b-value increases with positive pulse duration (t_0_) and decreases as the ratio of I_i_/P_i_, which decreases with stand-off distance. The b-values for the tabulated data were determined. Karlos et al.^27^ analyzed b-values that define the shock wave. The b-value is an important parameter. It is related to the potential work the shock wave can do on objects and people in the path of propagation. The larger the b-value, the smaller the relative amount of possible energy transfer from a pressure wave to an object in the path. The b-value is large close to the center of the explosion and decreases with stand-off distance.

### Temporal and spatial propagation of a shock wave

The Friedlander description of the shock wave was used to plot the temporal and spatial change in the wave for R = 2–10 m with a 15 kg TNT charge (Z = 0.8–3.4 m/kg^1/3^). Four distances were chosen to show the temporal and spatial pressure waveshape as the shock propagates. The wave shape of pressure at R = 1.97, 3.95, 5.92 and 8.38 m was determined for a 15 kg TNT hemispherical charge, representing Z = 0.8, 1.6, 2.4 and 3.4 m/kg^1/3^. The Z = 1.6 m/kg^1/3^ (3.95 m) condition is a typical stand-off distance for blast testing of armored passenger-vehicles with a 15 kg charge^5^. Other tests are conducted with Z = 0.8 m/kg^1/3^ (2 m) using a shaped charge.

The spatial shape of the shock wave is not commonly discussed in the literature. Friedlander waves were determined for small increments (0.1 m) of standoff distance over time up to 10 ms. The family of curves provide the spatial shape of the shock wave as snapshots in time. The spatial shape behind the shock front was determined at t = 0.78, 2.75, 5.85 and 10.85 ms representing the arrival time of a 15 kg TNT explosion at 1.97, 3.95, 5.92 and 8.38 m, respectively. The spatial distribution decays behind the shock wave front and has a cross-over distance (x_0_) that corresponds with t_0_. While the spatial decay near the explosion can be represented by an exponential decay. The decay profiles at 3.95–8.38 m cannot be represented by an exponential. Second-order polynomials fit the spatial decay above R = 3.95 m with a 15 kg explosion (Z = 1.6 m/kg^1/3^).

### 15 kg TNT hemispherical shock wave at 3.95 m (Z = 1.6 m/kg^1/3^)

The temporal and spatial wave shapes at 3.95 m were analyzed in detail for a 15 kg TNT hemispherical blast. The volume of the shock, surface area at the front and weight of the air compressed behind the wavefront were determined assuming an adiabatic process. The speed (u) of the compressed air behind the shock front and the density (ρ) of the compressed air were determine for adiabatic conditions. The air density was calculated with:

6 $$ \rho = \gamma {\text{P}}/{\text{u}}^{{2}} , $$

where γ = 1.401 is the ratio of specific heat for ambient air, P is the pressure and u is the velocity of the wave behind the shock front^9^. The wave properties were determined for 0.1 m increments behind the wave front, including the weight per unit surface area of the shock.

### 15 kg TNT hemispherical shock wave loading a vehicle

A 15 kg TNT hemispherical blast wave was analyzed with an armored passenger-vehicle at 4 m (Z = 1.6 m/kg^1/3^) near side and 6 m far side. The force on the near-side and far side of the vehicle were determined using the side area of the vehicle and the Friedlander pressure. An armored SUV was analyzed with a mass (m_v_) of 3,773 kg and side area (A_v_) of 4.2 m^2^. The force (F) on the near side of the vehicle was the product of the shock wave pressure and lateral surface area with F = P_r_^^^A_v_. The reflected pressure (P_r_^^^) was used on the near side facing the explosion with a peak of 1,977 kPa and b = 7.26. The pressure at first contact was used and gives an upper bound for the vehicle loading.

The width of the SUV is 1.97 m. The shock wave propagated over and under the vehicle eventually loading the far-side at 6 m and arrival time of t_a_ = 6.00 ms. The incident pressure (P_i_^^^) was 182 kPa and b = 2.15. The force was in the opposite direction for the far-side loading. The vehicle was assumed at rest when the shock wave contacted the near-side at t_a_ = 2.83 ms. The force accelerated the vehicle laterally with an opposing force at 6.00 ms. The lateral acceleration (a_v_) was:

7 $$ {\text{a}}_{{\text{v}}} = ({\text{F}} - {\text{km}}_{{\text{v}}} )/{\text{m}}_{{\text{v}}} = ({\text{P}}_{{\text{r}}}\hat{}{{{\text{A}}_{{\text{v}}} }} - {\text{P}}_{{\text{i}}}\hat{}{{{\text{A}}_{{\text{v}}} }} - {\text{km}}_{{\text{v}}} )/{\text{m}}_{{\text{v}}} , $$

where P_r_^^^ and P_i_^^^ have different arrival times and k = 0.3 is ground friction used to decelerate the vehicle to rest. A step-forward integration was conducted with 0.1 ms timestep to determine the rigid body velocity (v_v_) and displacement (s_v_) of the vehicle.

The side area of the SUV is greater behind the vehicle cg than in front with a ratio of 58% behind and 42% forward. The shock-wave causes yaw rotation with more displacement of the rear wheels than the front as the cg displaces laterally. The fore-aft placement of the explosion is another factor. Charges are often setup perpendicular to the B-pillar or center of the rear door, so the pressure causes vehicle yaw with differing offset from the cg. The yaw of the vehicle was determined from the moment about the cg. The yaw rotational acceleration (ω) was:

8 $$ \omega = \left[ {{\text{d}}\left( {{\text{A}}_{{\text{r}}} {-}{\text{A}}_{{\text{f}}} } \right){\text{P}}_{{\text{r}}} - {\text{m}}_{{\text{v}}} {\text{kr}}} \right]/{\text{I}}_{{\text{y}}} , $$

where A_r_ = 0.58A_v_ is the side area rear of the cg and A_f_ = 0.42A_v_ is the area forward, d is the moment arm of the differential force, r is half the wheelbase for ground friction to stop the rotation and I_y_ is the yaw moment of inertia for the vehicle. For the SUV, I_y_ was 7,800 kgm^2^, r = 1.42 m and d = 1.2 m. A step-forward double integration was conducted with 0.1 ms timestep to determine the yaw angle (θ) change of the vehicle.

The calculations provide a first-order determination of vehicle motion. More complicated simulations can add precision to the shock-wave propagation around the vehicle, deformation of the near-side sheet-metal and structures and the overall vehicle dynamics. The incident pressure on the roof and underbody was determined at 4.5 m. The reflected pressures under the vehicle are complex as the shock passes the vehicle. The incident pressure is a lower bound on the floor and roof loading.

As the shock wave passes the vehicle, the floor is accelerated up and the roof down. The forces cause local deformation of sheet metal attached to vehicle structures. For the floor, the force is related to the segment area (A_s_) of floor. The vehicle structures deform locally in proportion to the mass (m_s_) of the segment and stiffness (j) of the connections of the segment of floor to surrounding vehicle structures. The local acceleration (a_s_) depends on the segment mass (m_s_), stiffness (k) of floor connections and segment area (A_s_). Local displacements can involve high velocity toward the occupant with the segment springing back into position after the pressure passes. Local deformations load the occupant’s feet in contact with the floor. Other areas of the interior can be deformed in body regions of an occupant.

An underbody analysis was conducted. It used a factor ξ increasing the incident pressure of 350 kPa due to reflections off the ground and underbody of the vehicle as the shock wave propagates. A value of ξ = 1.3 was used. The area of the floor (A_s_) under the near-side occupants was 1 m long and 0.2 m wide for the shock wave arriving at 4.5 m and 3.52 ms. The segment area of floor was 0.2 m^2^. The floor was composed of 16-guage sheet metal with weight of 12.2 kg/m^2^ and thickness of 1.52 mm. The weight (m_s_) of the segment was 2.45 kg. The segment was part of the floor structure with a connected stiffness (j) of 2,500 kN/m. A step-forward calculation was made for the floor acceleration (a_s_), velocity (v_s_) and displacement (x_s_). The floor acceleration (a_s_) is:

9 $$ {\text{a}}_{{\text{s}}} = \left( {{\text{P}}_{{\text{i}}} {\text{A}}_{{\text{s}}} - {\text{jx}}_{{\text{s}}} } \right)/{\text{m}}_{{\text{s}}} $$

where P_i_ is the adjusted pressure and x_s_ is the floor displacement upward, which was obtained by double integration of the floor acceleration. If the occupant’s feet are on the segment of the floor that is rapidly accelerated, injury can occur by the floor deformation as the shock wave passes under the vehicle. An analysis of roof deformation was not made. The analysis did not determine vehicle lift because of the small differential force on the roof and underbody.

### Injury and death to armored passenger-vehicle occupants and ground personnel

There is a vast literature on blast overpressure injuries and death. The early work of Richmond et al.^52,53^, Bowen et al.^54^ and White et al.^55^ describe many experiments with shock wave exposures causing injury and death in experimental animals. Bass et al.^56^ analyzed the body of experiments and provided tolerance curves for incident and reflected pressure based on the pulse duration of the exposure. There are risk curves for injury threshold, 1% death, 50% death and 99% death. The pressure duration is the positive pulse duration (t_0_) of the empirical data for explosions with varying stand-off distance and charge weight. The pulse duration was matched with the tolerance data providing curves for the risk of injury and death with standoff distances of 1.0–49.3 m with 15 kg TNT and 1.9–32.5 m with 100 kg TNT explosions. For the reflected pressure tolerance, the 50% lethality data are included from Bowen et al. (1968). The curves evaluate risks for injury and death to ground personnel at varying distance from a 15 kg and 100 kg TNT hemispherical explosion.

The risk for injury and death for occupants inside an armored passenger-vehicle is often assessed using ATD (anthropometric test devices) in blast tests. The Hybrid III dummy has more than 100 channels of measurements related to injury risks for the head, neck, shoulder, chest, pelvis and legs^6,7^. The leg instrumentation evaluates the knee and ankle joints, the femur and tibia. For an occupant on the near-side of an explosion, the shock wave on the side of the vehicle deforms sheet metal and structures. The risk can be high if the occupant’s head or shoulder are against the near-side interior. Measurements of sound pressure are made inside the vehicle to assess risks for hearing injury or more severe injury if pressure breaches.

For vehicle occupants, the shock-wave and fragments propagate into and across the vehicle. The shock-wave loads the side of the vehicle deforming sheet metal and vehicle structures and displacing the vehicle by rigid-body motion. The wave passes the vehicle loading the roof and floor. The dynamics have different effects that create risks for the occupants. Blast tests are often conducted without an ATD and design engineers need measurements of vehicle dynamics that inform whether there may be risks of injury. The following injury risks need to be considered for occupants of armored passenger-vehicles:

Rigid-body motion of the vehicle

- Side pressure accelerates the vehicle causing a lateral velocity (delta V_v_) and yaw. The occupants displace inside and can be injured by secondary impacts on the interior if the delta V and yaw are sufficient.
- Underbody pressure lifts the occupant compartment raising the seat cushions with potential flexion-compression fractures of the lumbar spine.

Local intrusion of the occupant compartment

- Side pressure flattens sheet metal and frame structures with local intrusion potentially injuring the occupant by deformation of the body or accelerating the occupant into secondary impacts on the interior.
- Side deformation stresses seams around the doors and glass with potential breach of the occupant compartment with blast over-pressures loading the occupant.
- Underbody and roof pressure deforming the floor and roof inward with parts of the body, such as the lower extremities, in contact or near the intrusion.

Blast fragments

- Fragments can penetrate the side structures, enter the interior and impacting the occupants causing penetrating injury.

Body parts in contact with the floor, side and roof of the interior of the vehicle are exposed high accelerations as the pressure loads vehicle structures. Feet on the floor, shoulder against the side interior or head on side window involve interaction with accelerating structures that can cause injury. For occupants with a shoulder or head against the side interior, the local deformation of the door, glass and interior trim is specific for the vehicle and armor. It depends on the integration of the body armor and seams around the door frame and glass. The guidance from simple segmental models, like the floor analysis described here, gives insight on potential injury risks. It directs blast testing to determine biomechanical responses with an instrumented ADT in the vehicle.

### Injury by local intrusion: deformation and velocity of the body loading

As the shock wave deforms the perimeter of the occupant compartment, portions of the perimeter locally deform inward. The occupant typically has a gap to the interior, which isolates them from the initial deformation of the interior. If the intrusion closes the gap and impacts the occupant, the body is deformed and displaced by the load. The intrusion is y_i_, the gap to the occupant is y_g_, the displacement compressing the body is y_d_ and acceleration displacing the body is y_k_. The intrusion displacement is:

10 $$ {\text{y}}_{{\text{i}}} = {\text{y}}_{{\text{g}}} + {\text{y}}_{{\text{d}}} + {\text{y}}_{{\text{k}}} $$

The dynamic loading involves energy transfer to the occupant in the form of deformation of the body (E_d_) and acceleration of the body causing a velocity change, which transfers kinetic energy (E_k_). The deformation energy (E_d_) is related to the force (F_i_) on the occupant from local intrusion with:

11 $$ {\text{E}}_{{\text{d}}} = {\text{F}}_{{\text{i}}} {\text{y}}_{{\text{d}}} = {\text{ m}}_{{\text{b}}} {\text{a}}_{{\text{b}}} {\text{y}}_{{\text{d}}} $$

where F_i_ is the force on the occupant from local intrusion and a_b_ is the acceleration of body region being deformed. The acceleration of the body is related to the intrusion velocity, with a_b_ = Δv_d_/ΔT, where Δv_d_ is the intrusion velocity deforming the body after closing the initial gap (y_g_) and ΔT is the duration of load. Energy is transferred by deforming the body:

12 $$ {\text{E}}_{{\text{d}}} = \left( {{\text{m}}_{{\text{b}}} /\Delta {\text{T}}} \right)\Delta {\text{v}}_{{\text{d}}} {\text{y}}_{{\text{d}}} $$

Since the occupant is at rest before the explosion, the delta velocity of body deformation is just the velocity of deformation (Δv_d_ = v_d_) and the delta duration is merely the duration of loading (ΔT = T), so that:

13 $$ {\text{E}}_{{\text{d}}} = \left( {{\text{m}}_{{\text{b}}} /{\text{T}}} \right){\text{v}}_{{\text{d}}} {\text{y}}_{{\text{d}}} $$

The relationship shows that the energy transfer to body deformation is proportional to v_d_y_d_, which is the intrusion velocity compressing the occupant times the displacement deforming the body. For high-speed deformation, injury is related to viscous mechanism (v_d_y_d_), which is related to the product of the velocity of deformation times the amount of deformation^57^. The product is related to the strain times strain rate (ε*dε/dt) at the tissue level. For low-speed deformation, injury is related to crush mechanism, which is the tolerable compression of the body or the strain at failure (ε) at the tissue level.

The energy (E_k_) related to a velocity (v_k_) change by accelerating the body region is kinetic energy with:

14 $$ {\text{E}}_{{\text{k}}} = 1/2{\text{m}}_{{\text{b}}} {\text{v}}_{{\text{k}}}^{{2}} , $$

where m_b_ is the mass and v_k_ is the velocity of the body region. The kinetic energy can involve secondary impacts on the interior of the occupant compartment with potential injury.

### Injury related to v_d_y_d_

A series of blunt abdominal impact tests demonstrate the biomechanics of injury by dynamic deformation of the body. Lau et al.^58^ ran 20 anesthetized swine weighing 49.5 ± 2.0 kg on a Hyge sled. The delta V of the sled was 32 km/h (8.89 m/s). The abdomen was 0.25 m away from the lower rim of a steering wheel. One series of 8 tests involved a lower rim stiffness of 3.0 kN (stiff rim) and the other of 8 tests, a load-limiting rim with 1.0 kN stiffness (soft rim).

The stiff rim tests involved VC = 1.82 ± 0.36 m/s and abdominal compression of C = 42 ± 4% with all 8 animals having critical-fatal abdominal injury to the liver. The abdominal depth (D) was 27.2 ± 1.7 cm for the stiff-rim group and 26.3 ± 1.5 cm for the soft-rim group. The soft rim tests involved VC = 0.87 ± 0.12 m/s and abdominal compression of C = 34 ± 3%. Three of 8 animals had minor liver laceration and 5 had no injury. The same notation is used here as in the original study with V, the velocity of abdominal compression (v_d_) and C, the compression of the abdomen with C = d/D, with d, the deflection of the abdomen (y_d_) and D, the abdominal depth. The deformation of the abdomen (y_d_) and velocity of deformation (v_d_) are VC = v_d_y_d_/D, which relates the experimental results to the analysis conducted here.

## Results

Table 1 summarizes the incident and reflected pressure (P_i_ and P_r_), incident and reflected impulse (I_i_ and I_r_), shock wave velocity (U), arrival time (t_a_) and duration of the positive pressure pulse (t_o_) for various Z and R stand-off distances with a 15 kg TNT hemispherical explosion. The data comes from empirical relationships^8,12,17,43^. Many of the calculation are not possible or accurate close to the detonation center. The ratio of the incident to reflected pressure increases from the explosion center to a peak of 10.8 at Z = 0.2 m/kg^1/3^ and progressively drops to 2 at large distances. The detonation velocity of TNT is included. Data above Z ≥ 0.4 m/kg^1/3^ is assumed reliable.

**Table 1:** Shock wave characteristics with stand-off distance from empirical relationships of hemispherical blasts (from Kingery^8^, Kingery, Bulmash^12^ and Swisdak^17^).

Z (m/kg^1/3^)	Stand-off	Peak pressure			Time		Velocity (m/s)	Impulse	
	Distance* (m)	Incident (kPa)	Reflected (kPa)	Ratio Refl/Inc	Arrival (ms)	+ phase		Incident (kPa*ms)	Reflected (kPa*ms)
0.05	0.123	5,08,092	11,30,022	2.2	–	–	6950^	–	–
0.08	0.197	94,852	5,98,152	6.3	0.03	–	6534	5,400	1,49,882
0.1	0.247	54,821	4,65,251	8.5	0.04	–	5859	3,453	94,681
0.2	0.493	17,316	1,86,259	10.8	0.09	0.60	4003	911	25,968
0.4	0.986	6834	59,407	8.7	0.25	0.58	2544	432	8235
0.6	1.48	3612	27,485	7.6	0.48	0.94	1890	421	4463
0.8	1.97	2135	14,419	6.8	0.78	2.01	1478	496	2962
1.0	2.47	1357	8147	6.0	1.15	4.28	1197	583	2182
1.2	2.96	914	4898	5.4	1.61	5.44	1000	529	1713
1.4	3.45	645	3114	4.8	2.14	5.41	860	465	1403
1.6	3.95	475	2080	4.4	2.75	5.19	759	411	1184
1.8	4.44	362	1454	4.0	3.44	5.06	683	367	1022
2.0	4.93	284	1057	3.7	4.18	5.06	626	332	897
2.2	5.43	228	795	3.5	4.99	5.21	582	303	799
2.4	5.92	188	616	3.3	5.85	5.49	547	280	720
2.6	6.41	157	490	3.1	6.77	5.93	520	260	654
2.8	6.90	134	399	3.0	7.73	6.56	498	243	600
3.0	7.40	116	331	2.9	8.74	6.98	479	229	553
3.4	8.38	89.4	239	2.7	10.9	7.67	452	205	479
4.0	9.86	64.9	162	2.5	14.3	8.44	424	178	398
4.5	11.1	52.1	125	2.4	17.3	8.94	409	160	348
5.0	12.3	43.2	101	2.3	20.3	9.34	398	146	310
6.0	14.8	31.7	71.3	2.2	26.7	9.98	383	124	253
7.0	17.3	24.9	54.7	2.2	33.2	10.5	374	108	214
8.0	19.7	20.3	44.1	2.2	39.9	11.0	368	94.7	186
10.0	24.7	14.8	31.6	2.1	53.4	11.8	361	76.5	146
12.0	29.6	11.6	24.4	2.1	67.0	12.5	357	64.1	121
14.0	34.5	9.49	19.8	2.1	80.8	13.2	354	55.3	103
16.0	39.5	8.02	16.6	2.1	94.7	13.7	352	48.6	89.3
18.0	44.4	6.92	14.2	2.1	109	14.2	350	43.3	78.9
20.0	49.3	6.07	12.4	2.0	123	14.6	349	39.2	70.7
39.8	98.0	2.38	4.81	2.0	263	17.6	344	19.7	34.4
*with 15 kg TNT					^detonation velocity of TNT				

**Figure 1:**
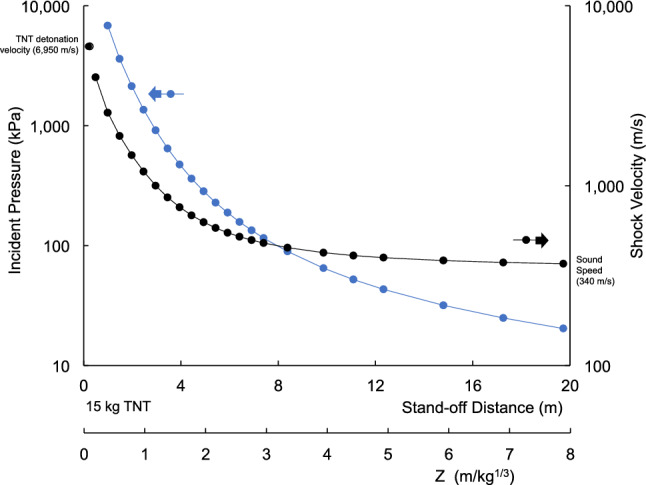
Incident overpressure and shock velocity with stand-off distance.

**Figure 2:**
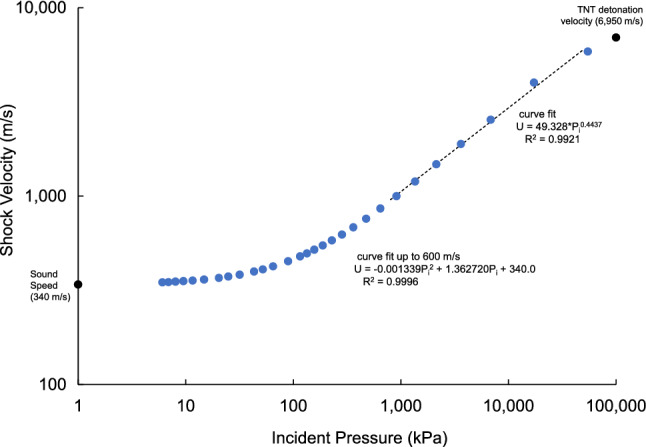
Shock wave velocity versus incident overpressure.

**Figure 3:**
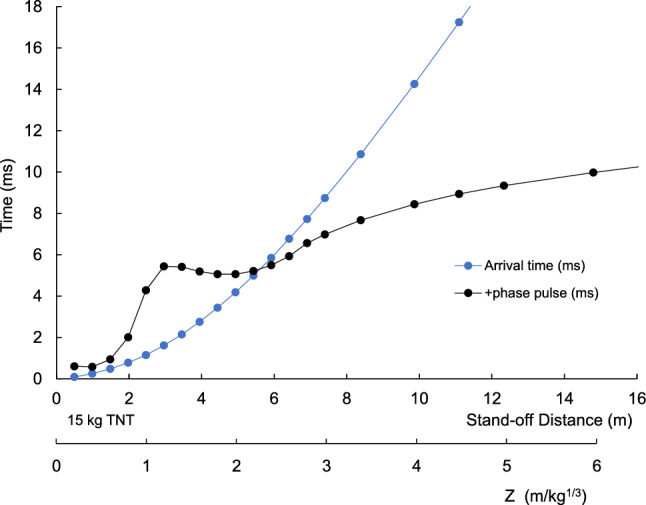
Shock wave arrival time (t_a_) and positive pulse duration (t_0_) with stand-off distance.

**Figure 4:**
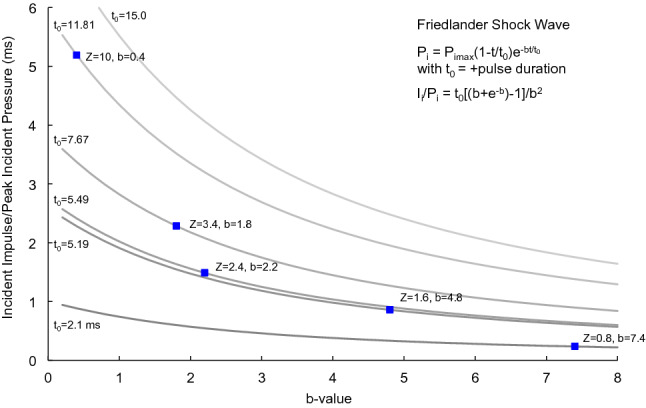
Friedlander shock wave b-value versus the + pulse duration (t_0_).

Figure 1 shows the incident pressure and velocity of the shock wave front at increasing stand-off distances. Both are plotted using logarithmic scales indicating large reductions with stand-off distance. Figure 2 shows the shock wave velocity increases with the incident pressure from 340 m/s in ambient air to the detonation velocity of TNT. Figure 3 shows the shock arrival time increase with stand-off distance. The positive duration of the pressure rises to a plateau and then increases after Z = 2 (6 m). Figure 4 shows b-values for the Friedlander equation as a function of the duration of positive pressure (t_0_) and ratio of incident impulse to pressure. The b-value for five stand-off distances is indicated for the different Z. As Z decreases, b increases toward the center of the explosion as t_o_ decreases. The b-value is 0.4 at a large distance from the blast center.

**Figure 5:**
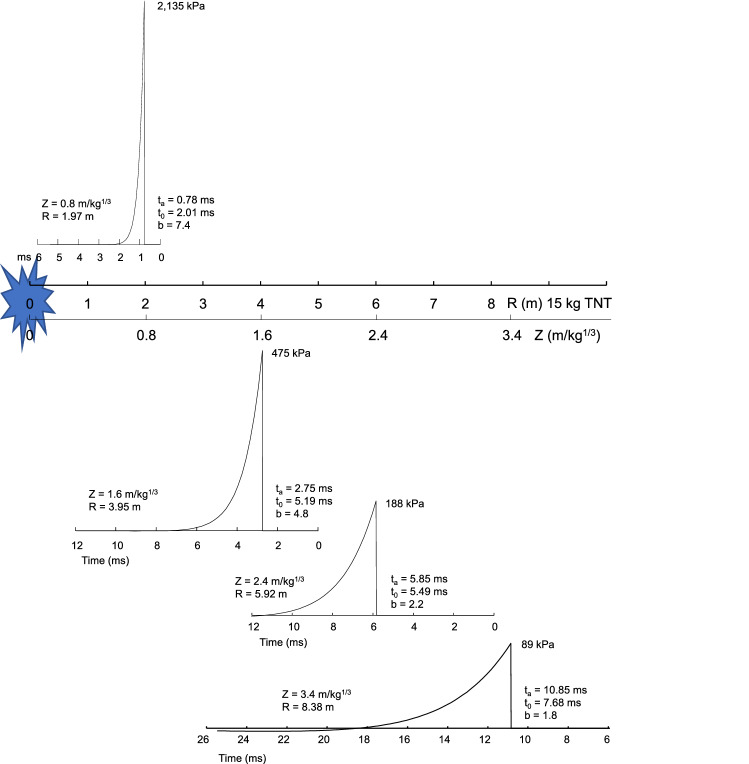
Shock wave characteristics versus time after arrival (t_a_) with stand-off distance.

**Figure 6:**
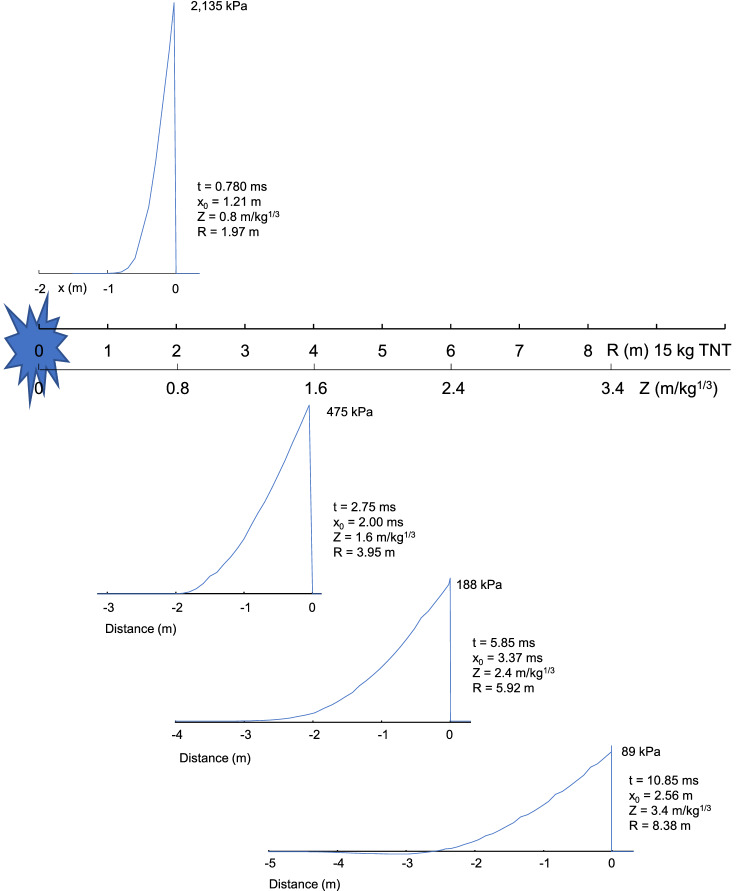
Shock wave characteristics versus distance behind arrival (t_a_) with stand-off distance.

Figure 5 shows the Friedlander wave shape at four stand-off distances from a 15 kg TNT charge. The plots show the temporal change in pressure. Near the explosion, the b-value is large causing a sharp decay in the pressure behind the wave front. The wave spreads in time and space. The incident pressure drops with distance and the b-value decreases indicating a more gradual decay behind the wave front. Figure 6 the spatial change in wave shape for the four stand-off distances at a fixed time with 15 kg TNT charge. The cross-over point is x_0_, which corresponds with t_0_ in the Friedlander equation.

### 15 kg hemispherical blast shock wave at 3.95 m (Z = 1.6 m/kg^1/3^)

**Figure 7:**
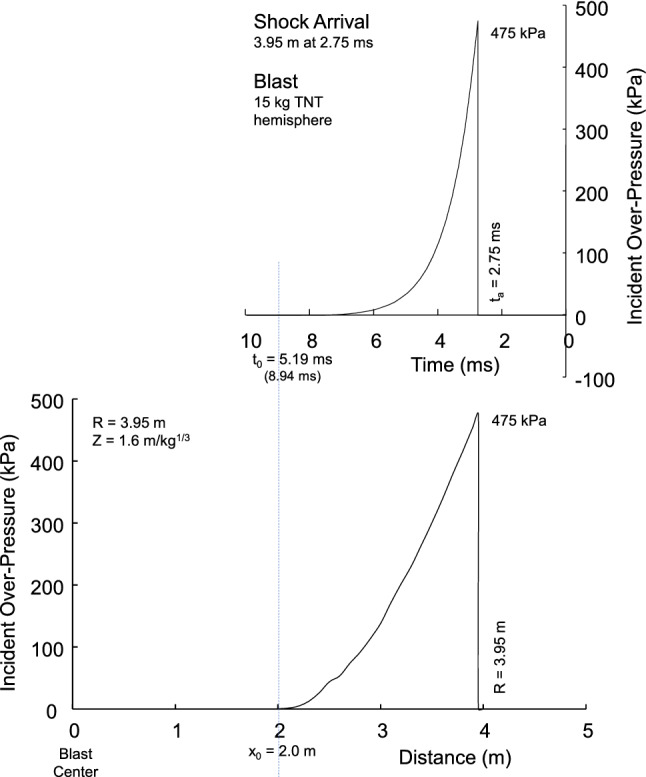
Time and special distribution of shock wave for 3.95 m stand-off distance with 15 kg hemispherical blast.

Figure 7 shows the temporal and spatial change in pressure for Z = 1.6 m/kg^1/3^, 3.95 m stand-off distance with a 15 kg TNT charge. The crossover time is t = 8.94 ms with t_a_ = 2.75 ms and t_0_ = 5.19 ms. The crossover distance is x_0_ = 2 m behind the shock front. The shock wave arrival and cross-over pressure were aligned. The broader spatial profile is consistent with the slower velocity at lower pressure than the peak.

**Figure 8:**
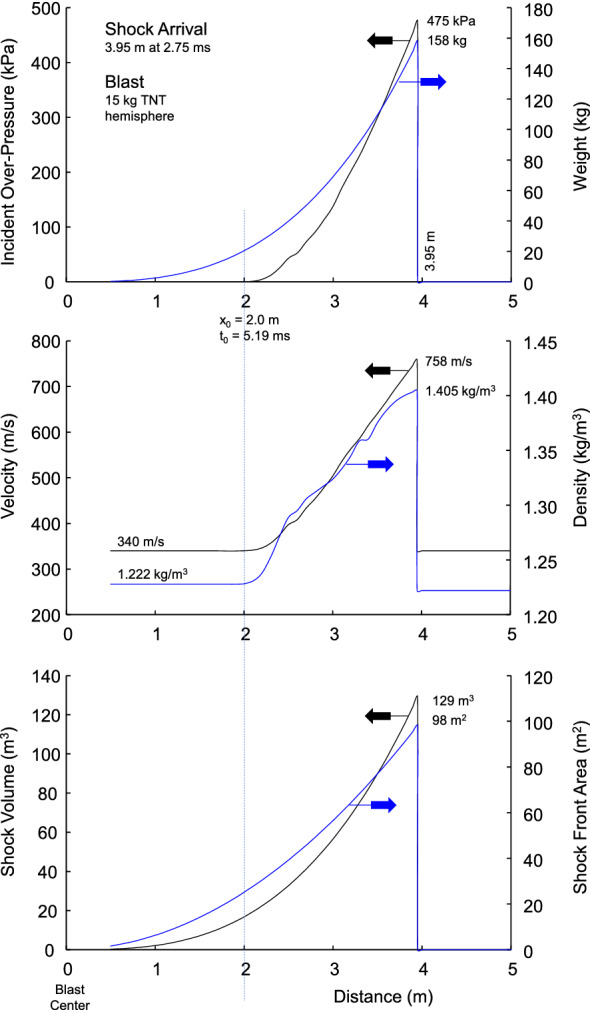
Shock wave characteristics for 3.95 m stand-off distance with 15 kg hemispherical blast.

Figure 8 shows more details on the shock wave at Z = 1.6 m/kg^1/3^, R = 3.95 m. The volume of air behind the shock front is 129 m^3^ at ambient pressure weighing 158 kg. The surface area at the wave front is 98 m^2^. giving a weight density of 1.61 kg/m^2^ in the outer shell at the wave front. The air has been accelerated forward by the explosion with progressively heavier layers of the blast wave up to the wave front. The air is compressed the most behind the wave front with a maximum density of 1.405 kg/m^3^ in this snapshot in time (t = 2.75 ms). The higher density is consistent with the higher velocity and pressure at the wave front. The velocity of the wave front is 758 m/s, which is consistent the 1.405 kg/m^3^ air density behind the shock front. The weight and density of layers behind the shock front decrease with decreasing wave speed. The speed decreases to the speed of sound (340 m/s) in ambient air.

### 15 kg TNT hemispherical shock wave loading a vehicle

**Figure 9:**
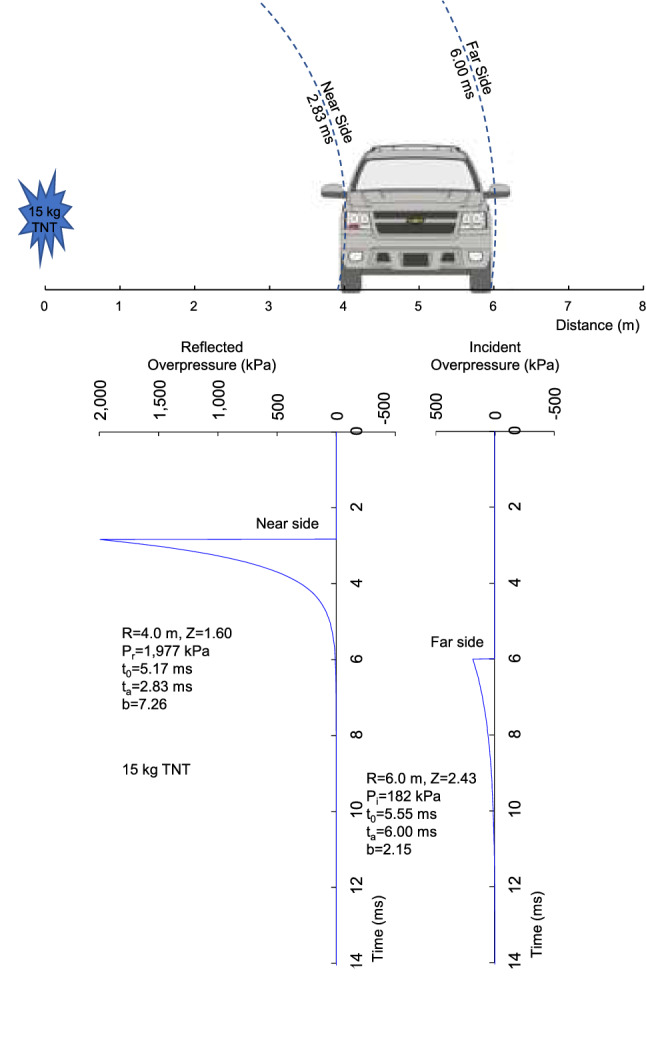
Shock wave loading of an armored passenger-vehicle with 4 m stand-off from 15 kg blast.

Figure 9 shows the near side of an armored vehicle at 4 m and far side at 6 m. A 15 kg TNT hemispherical explosion creates a shock wave that strikes the near side at t_a_ = 2.83 ms with a reflected pressure of 1,977 kPa. The Friedlander decay in pressure is shown at initial contact with the vehicle. The shock reaches the far side at t_a_ = 6.00 ms with an incident pressure of 182 kPa.

**Figure 10:**
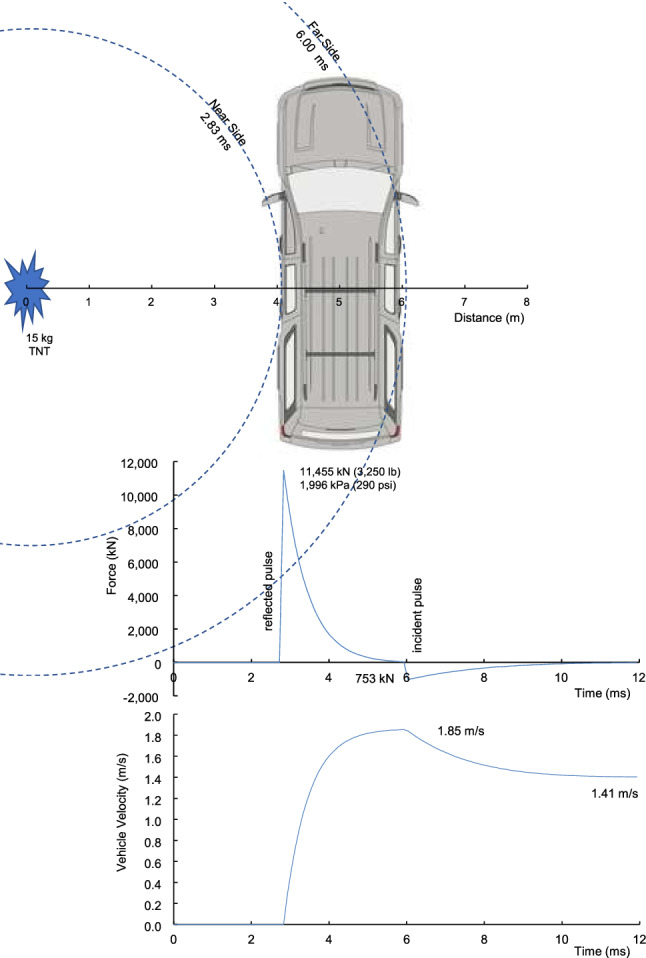
Force on the side of an armored passenger-vehicle and lateral velocity of the vehicle with 4 m stand-off from 15 kg blast.

**Figure 11:**
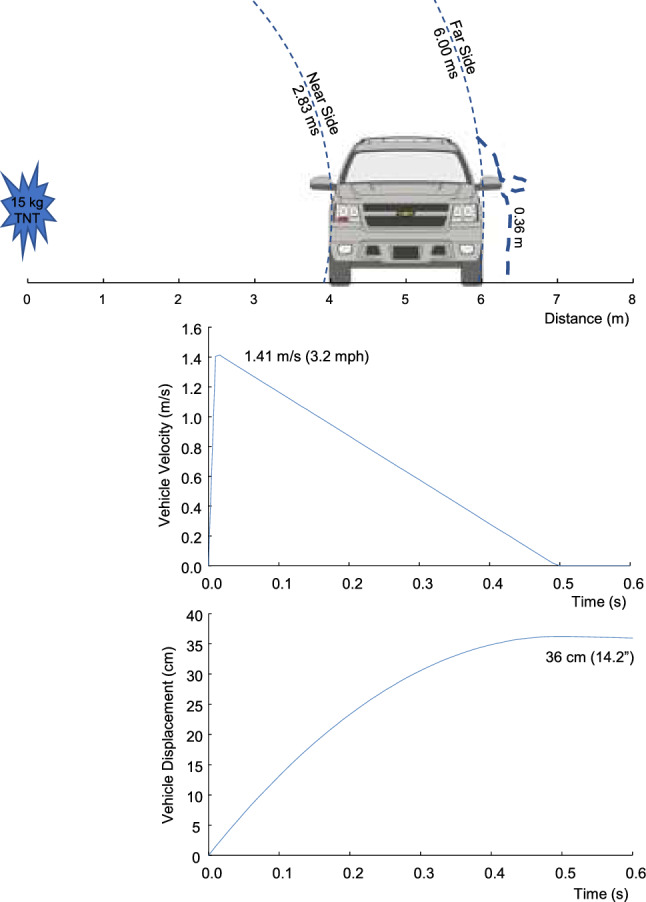
Lateral velocity and displacement of an armored passenger-vehicle with 4 m stand-off from 15 kg blast.

Figure 10 shows the lateral force on the vehicle with a near-side peak of 11,455 kN. The far side load occurs later and has a peak of 753 kN in the opposite direction. The force and vehicle mass were used to integrate for the lateral velocity of the vehicle. It reached a peak of 1.85 m/s at 6.00 ms and decreased by the opposing force on the far-side of the vehicle. By 12 ms, the lateral velocity was 1.41 m/s. Figure 11 shows the reduction in velocity by ground friction until the vehicle came to rest at 0.5 s. The lateral forces on the vehicle caused 36 cm displacement of the vehicle cg (center of gravity) at 0.5 s. The yaw angle was 7.8 deg at 0.25 s.

**Figure 12:**
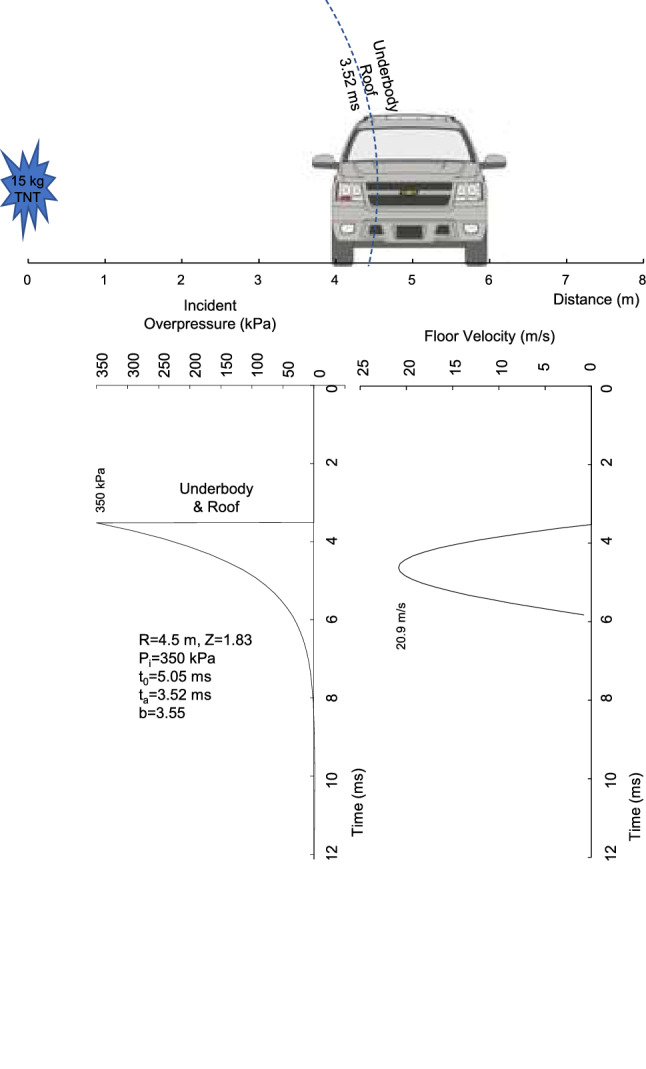
Underbody and roof loading of an armored passenger-vehicle with 4.5 m stand-off from 15 kg blast.

Figure 12 shows the incident pressure under and above the vehicle at 4.5 m with a peak of 350 kPa and arrival time of t_a_ = 3.53 ms. The shock wave front is at the underbody and roof loading the vehicle upward and downward. The dynamics of the floor deformation were analyzed. The peak load was 91 kN on the floor segment with ξ = 1.3. The force caused 20.9 m/s floor velocity at 4.62 ms and upward displacement of 3.2 cm at 5.82 ms. The floor sprang back as the pressure dropped with shock wave propagation beyond the underbody.

### Injury and death of passengers and ground personnel

The risk of injury and death for occupants inside an armored passenger-vehicle typically need to be assessed using ATD (anthropometric test devices) in blast tests. However, calculations of deformation of vehicle structures provide perspective on possible injury. For example, the floor deformation as the shock wave passes under the vehicle in Fig. 12 involves upward force on the floor. The model gives a maximum floor deformation of 3.2 cm, but the velocity is high at 20.9 m/s. The more the occupant’s feet are coupled to the floor, the more the high velocity movement loads the feet with potential injury to the ankle, tibia and leg.

**Figure 13:**
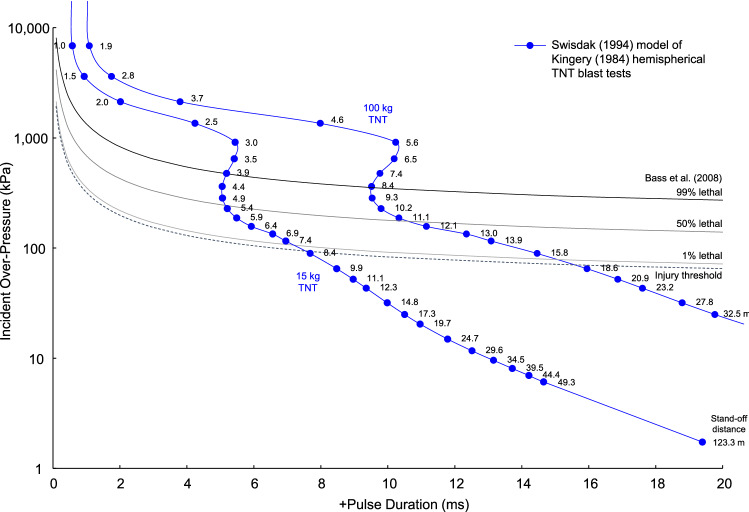
Injury and fatality risks with stand-off distance from incident shock waves.

Figure 13 shows the injury threshold and 1%, 50% and 99% risk of death with incident pressure at stand-off distances from the center of detonation. The blue dots represent empirical results for a 15 kg and 100 kg hemispherical TNT explosion. The background lines show the human tolerances based on many experiments analyzed by Bass et al. (2008). The two datasets are connected by the pulse duration of the over-pressure. The standoff distance is 1.0–49.3 m with 15 kg TNT explosion and 1.9–32.5 m with 100 kg TNT explosion.

**Figure 14:**
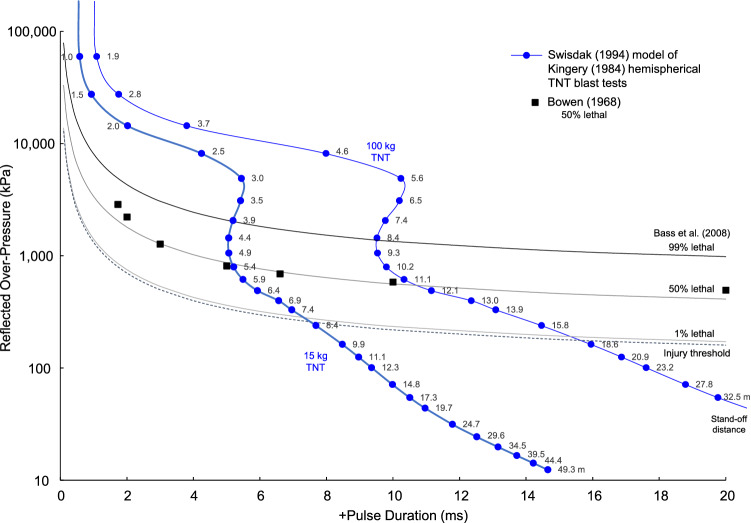
Injury and fatality risks with stand-off distance from reflected shock waves.

The Kingery-Bulmash empirical curves cross the tolerance lines and can be used to evaluate risk for injury and death to ground personnel at varying distance from a 15 kg and 100 kg TNT hemispherical explosion. Figure 14 shows the threshold for injury and 1%, 50% and 99% risk of death with reflected pressure at stand-off distance of 1.0–49.3 m with 15 kg TNT explosion and 1.9–32.5 m with 100 kg TNT explosion. The 50% lethality data are included from Bowen ^54^. A 15 kg charge posed a 99% risk of death at 3.9 m, 50% risk at 5.2 m, 1% risk at 7.8 m and injury threshold at 8.2 m. A 100 kg charge posed a 99% risk of death at 8.5 m, 50% risk at 11.6 m, 1% risk at 17.3 m and injury threshold at 18.0 m. Based of Z, the 99% risk of death at Z = 1.7 m/kg^1/3^, 50% risk at Z = 2.3 m/kg^1/3^, 1% risk at Z = 3.4 m/kg^1/3^ and injury threshold at Z = 3.6 m/kg^1/3^.

### Injury related to v_d_y_d_

**Figure 15:**
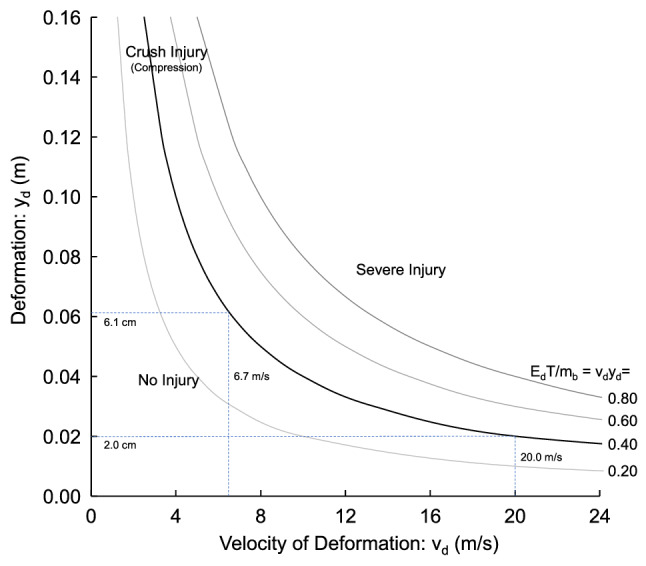
Iso-energy curves related to the intrusion velocity loading and deformation of the body.

Figure 15 shows iso-energy transfer curves for intrusion deforming a body region of the occupant. The curves relate the velocity (v_d_) of deformation to the deformation (y_d_) of the body with equal energy transfer (E_d_). Since the occupant is at rest prior to the explosion, Δv_d_ is equal to v_d_ and the ΔT is merely the duration of the deformation, or T. Each curve is equal energy transfer. The bold line is for E_d_T/m_b_ = v_d_y_d_ = 0.4 (m/s)m was empirically found to separate non-injury from injury exposures. If intrusion deforms the occupant at 6.7 m/s, 0.4 (m/s)m energy transfer occurs with 6.1 cm deformation of the body. If greater compression occurs, more energy is transferred. Injury is related to energy transfer. The greater the energy transfer, the greater the risk of injury. The 0.4 (m/s)m energy curve indicates that high intrusion velocity has similar energy transfer as the 6.7 m/s impact if the displacement is lower. At 20 m/s deformation velocity, similar energy is transferred at 2.0 cm deformation.

**Figure 16:**
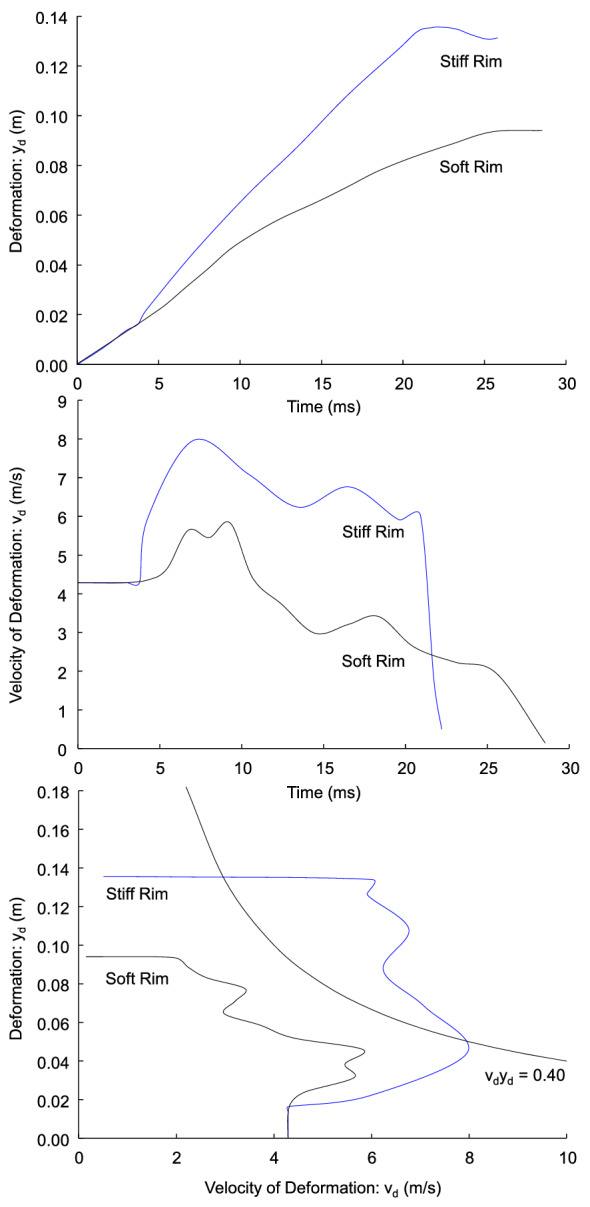
Abdominal compression tests with stiff and soft rim (based on Lau et al.^58^).

Figure 16 (top) shows the deformation of the abdomen in the stiff-rim and soft-rim sled tests from Lau et al.^58^. After the gap is closed, the rim deforms the abdomen increasing compression. Figure 16 (center) shows the velocity of deformation in the stiff- and soft-rim tests. With the soft-rim, wheel deforms the abdomen at lower velocity because there is load-limiting deformation of the wheel rim. The amount of velocity and compression is greater in the stiff rim tests, so VC is higher. Figure 16 (bottom) shows the v_d_y_d_ responses in the stiff- and soft-rim tests as a function of the velocity of deformation (v_d_) and abdominal deformation (y_d_). The graph includes a curved line with for E_d_T/m_b_ = v_d_y_d_ = 0.4 (m/s)m. The tests with the rigid-rim exceed the iso-line of constant energy absorbed by body deformation; whereas, the soft-rim tests remain below the iso-energy line. The experimental data shows the importance of controlling interior panel deformation that lowers v_d_y_d_ and the risk of injury.

## Discussion

### Rigid body displacement of the armored passenger-vehicle

The impulse from the 15 kg blast at 4 m loads the side of the vehicle and causes 1.85 m/s lateral velocity (delta V) at 5.93 ms and 36 cm displacement at 0.50 s based on rigid body mechanics. This level of delta V is well below the threshold for severe injury based on field accident data for car occupants. Viano and Parenteau^59^ reported near-side occupants had a risk of 0.083% ± 0.057% for severe injury in < 16 km/h (4.4 m/s) delta V side impacts using a large database of crashes investigated by the U.S. government. Far-side occupants had a risk of 0.022% ± 0.022%. The delta V is below the threshold for deployment of side curtains and torso airbags in modern vehicles. The delta V is also below the interior head impact testing that is part of U.S. safety standards. FMVSS 201 involves a free-motion head-form impact on the interior at 5.4 m/s (12 mph) near an inflatable restraint and 6.7 m/s (15 mph) at other locations on the interior above the beltline^60^. There is no concern for occupant safety in this severity of delta V and displacement with 15 kg explosion at 4 m. The side area of SUVs is greater behind the vehicle cg. The shock-wave causes yaw rotation with more displacement of the rear wheels than the front. For the charge perpendicular to the center of the rear door, the moment on the vehicle caused 7.8 deg yaw rotation. This level of rotation is small and does not cause injury.

### Flattening the near-side of the vehicle

The side blast involves 11,455 kN force at the arrival of the shock wave at 4 m with 15 kg. This is related to a reflected pressure of 1977 kPa and side area of 4.2 m^2^ for the SUV. The high force flattens sheet metal covering the vehicle frame. It also acts to flatten the convex curvature of the vehicle door below the beltline (base of the side windows), which is closer to the charge than the roof line or sill. The flattening causes the door frame to pull away from the door ring at the top of the window frame. This is something seen in side impact testing with the barrier face loading the area below the beltline.

The shock-wave pressure deforms the vehicle frame of the occupant compartment. The deformation stresses side structures and seams around closures on the vehicle. The pressure can breach the door and window frames, enter the occupant compartment and load the occupant. The integration of door and window seams is important to prevent stress concentrations. Deformation of the side interior loads the head, shoulder, torso and hip of the near-side occupant if they are near the side interior. Biomechanical tests show that impact velocity and energy are needed to injure the shoulder. Tests at 5.8–8.8 m/s impact velocity result in shoulder injury with no injury at lower speeds^61,62^.

With charges close to the vehicle, fragments of the explosion load the side of the vehicle. The armor is designed to prevent fragments entering the occupant compartment. In blast tests, sheet metal is often placed on the near-side interior to “witness” fragment penetration. Pressure measurements in the vehicle are used to evaluate if the shock wave enters the occupant compartment with risks for ear injury or more severe trauma at higher pressures.

### Forces on the underbody and roof

As the shock wave passes under and over the vehicle, forces act to displace the vehicle and accelerate segments of the occupant compartment inward. The rigid-body lifting of the vehicle loads the pelvis upward stressing the lumbar spine. The biomechanics of upward acceleration of the pelvis has been widely studied with injury criteria to assess risks for lumbar injury^63,64^.

When the shock wave passes the near-side frame rail, forces act up of the sheet metal floor under the occupant’s feet. A segment of floor is accelerated up. The displacement depends on the mass and area of floor involved and the attachment to surrounding structures. SUVs have lateral braces between the longitudinal frame rails that support the sheet-metal floor. The vehicle can be a body on frame or integrated unibody, which changes the integration of the floor to surrounding structures. The dynamics of the floor can involve high upward velocity. The risk of leg injury depends on the velocity and displacement of the floor. Biomechanical testing has shown the magnitude of velocity and displacement causing injury to the lower legs. Impact tests at 7.2 m/s resulted in no injury and impacts at 9.9–11.6 m/s resulted in calcaneal and tibial fractures^62^. While the floor velocity is high in the analysis, the displacement is low limiting the energy transfer to the feet. The analysis shows the need for additional investigation of floor structures.

### Details of 3.95 m standoff with 15 kg explosion

At this stand-off distance the shock wave is traveling 758 m/s and there is 158 kg of air packed behind the shock front. The density is 1.408 kg/m^3^ compared to 1.222 kg/m^3^ at ambient pressure. The surface area of the shock is 98 m^2^ giving a weight density of 1.61 kg/m^2^. Figure 13 shows that ground personnel standing 4 m from the explosion have a 99% risk of fatality. The frontal area of a 50^th^ male chest is 0.131 m^2^ based on GEBOD dimensions^65^. The outer 15 cm layer of the shock wave has a weight of 0.0265 kg for this area and depth. The kinetic energy of the mass of compressed air is 7,606 J since the shock wave is traveling 758 m/s. This is an enormous energy loading the chest. Obviously, the compressed air does not act as a rigid body as it loads objects in the path of propagation, but the loading is at very high velocity.

Axelsson and Yelverton^66^ studied complex blast waves and found the velocity of chest loading was 3–4.5 m/s at the injury threshold, 8–12 m/s at 1% lethal and 12–17 m/s at 50% lethal. The study shows the importance of the velocity of deformation. The viscous mechanism combined the velocity and extent of deformation showing it was more predictive of soft-tissue injury because of its relationship to energy absorbed by the body with high-velocity loading. Yelverton^67^ analyzed chest injury with shock-wave loading and correlated injury with over-pressure, which provided early guidance on possible risks. Occupants in an armored passenger-vehicle are protected by the frame and sheet metal covering, which reflects the shock wave isolating the occupants from the over-pressure.

### Injury related to E_d_ ~ v_d_y_d_ and E_k_ ~ v_k_^2^

The shock wave deforms the perimeter of the occupant compartment with portions of the perimeter locally deforming into the occupant. The dynamic loading involves energy transfer to the occupant in the form of deformation of the body (E_d_) and velocity of the body giving it kinetic energy (E_k_).

Figure 14 shows iso-energy transfer curves for intrusion loading the occupant. The curves relate the velocity (Δv_d_ or v_d_) of loading to the deformation (y_d_) of the body with equal energy transfer. Each curve is equal energy transfer with the duration ΔT equal to T. For example, the bold line is for E_d_T/m_b_ = 0.4 (m/s)m. The 0.4 energy curve indicates that high intrusion velocity has similar energy transfer as the 6.7 m/s impact if the displacement is lower. At 20 m/s intrusion velocity, similar energy is transferred at 2.0 cm displacement.

The iso-energy curves can be viewed as curves of equal injury risks. The risk increases as the energy transfer increases to a higher iso-energy curve, as shown in Fig. 15. The measurement of intrusion of the occupant compartment is useful in blast tests and accelerometers are available with 100,000 g dynamic range. The accelerometer is attached to the interior of the vehicle at areas where occupant injury needs evaluation. The transducer measures the local acceleration in a blast test. The acceleration can be integrated for the velocity of intrusion and double integrated for displacement of the interior. The data can be interpreted with Fig. 14. Occupant injury at high speeds of loading is related to a viscous mechanism^57^. At low velocities of deformation, compression of the body causes crushing injuries. As more compression of the body occurs, more energy is transferred and injury risks increase. As greater deformation velocity occurs, more energy is transferred and injury risks increase. A design goal is to reduce the product of v_d_y_d_.

The floor intrusion analysis found an intrusion velocity of 20.9 m/s and 3.2 cm displacement. If there is 1 cm thickness of compressible carpet on the floor, there is 2.2 cm displacement of the foot. The response is almost on the 0.4 iso-energy curve indicating it is at the threshold for injury. There are risks to the ankle joint from deformation and to the lower extremity from upward velocity (v_k_). The risk of injury is lowered by reducing the velocity and displacement of the floor.

### Friedlander shock wave

The shape of the pressure behind the shock front is defined by an exponential decay in pressure based on Friedlander^51^. There have been a number of studies looking at the adequacy of the Friedlander profile. Rigby et al.^68,69^ analyzed the Friedlander equation with experiments showing differences as the pressure falls below 50% of the peak. Other studies show differences at low pressure during the tail-off in pressure^70^.

### Limitations

There are a number of limitations for the type of analysis conducted here. First, the Kingery-Bulmash and Swisdak relationships have known limitations close to the detonation^19,27,32,39^. Karlos et al.^27^ found that scaled parameters become inaccurate at low Z < 0.20 m/kg^1/3^. The analyses here involve Z = 0.8 m/kg^1/3^ and above, which is away from the near-detonation effects. They do not influence the risk assessment for the armored passenger-vehicle occupants. For car occupants, the closest stand-off distance was Z = 0.8 m/kg^1/3^. For ground personnel, the effective pressure for 99% fatality is Z = 1.6 m/kg^1/3^. There is no hope for survival with personnel closer to the detonation. Second, the Friedlander wave-shape was used to define the shock wave. The wave shape has been found to match experimental data^18^ and over-estimate pressures in the tail of the shock wave at pressures below 50% of the peak^71^. Third, the analysis is based on TNT explosions of an armored passenger-vehicle and ground personnel. Many different types of explosives are used today, so the equivalent amount for TNT needs to be determined to calculate shock-wave characteristics using Z. Scale factors are known for different explosives, although there are some difficulties with determining TNT equivalence^72–74^. Fourth, Fig. 10 shows the hemispherical wave contacting the near-side of the vehicle. The analysis for the rigid body displacement assumed a plane wave with characteristics of the initial shock-wave contact. The plane-wave analysis provides an upper bound on the vehicle dynamics, since pressure drops with distance to the front and rear of the vehicle. More complicated analyses can be conducted with CFD methods. There are reviews of factors affecting blast-wave interaction with objects^75^. Fifth, the determination of injury risks for occupants of armored passenger-vehicles is complex and depends on the age, gender, height, weight and other occupant characteristics. The biomechanical tolerances presented here are for the 50% male Hybrid III. Tolerances for the 5th female and 95th male Hybrid III are available and testing can be conducted with the 5th female and 95th male Hybrid III dummies^6,7^. Sixth, the determination of injury risks with an ATD involves measurements of dummy responses, including acceleration, force, moment and displacement. The measurements are made in a blast test and compared with tolerance data to interpret risk. It is useful to have guidance from the rigid-body velocity of the vehicle and the inward velocity of the occupant compartment from tests without a dummy to judge whether serious injury may occur in an exposure and vehicle. This type of velocity analysis can direct vehicle modifications, which can be verified in blast tests with a Hybrid III dummy. Seventh, the determination of the shock wave characteristics at 3.93 m standoff distance assumed adiabatic conditions. Dewey ^20^ showed elevated temperatures behind the shock front at this distance, which was not part of the analysis conducted here.

## Data Availability

All data generated and analyzed during this study are included in the published article. The sources for blast wave characteristics are cited in the paper. Any additional information on the step-forward calculations in Excel can be requested of the author at dviano@comcast.net.
